# A case of myeloperoxidase anti-neutrophil cytoplasmic antibody (MPO-ANCA)-associated glomerulonephritis and concurrent membranous nephropathy

**DOI:** 10.1186/1471-2369-14-73

**Published:** 2013-03-28

**Authors:** Michiko Shimada, Takeshi Fujita, Norio Nakamura, Ikuyo Narita, Yuko Shimaya, Reiichi Murakami, Hideaki Yamabe, Hiroshi Osawa, Ken Okumura

**Affiliations:** 1 Graduate School of Medicine, Division of Cardiology, Respiratory Medicine and Nephrology, Hirosaki University, 5 Zaifu-cho, Hirosaki, 036-8562, Japan; 2 Graduate School of Medicine, Community Medicine, Hirosaki University, 5 Zaifu-cho, Hirosaki, 036-8562, Japan

**Keywords:** MPO-ANCA, Crescentic glomerulonephritis, Membranous nephropathy

## Abstract

**Background:**

Myeloperoxidase anti-neutrophil cytoplasmic antibody-associated glomerulonephritis (MPO-ANCA-GN) and concurrent membranous nephropathy (MN) are very rare combination. Their causal relationship has been suggested, but not determined.

**Case presentation:**

A 73-years-old male with 5-year history of proteinuria underwent an operation for his sigmoid colon cancer. Seven months later, he was referred to a nephrology division due to an exacerbating renal function and hypoalbuminemia. Laboratory examination revealed positive MPO-ANCA in the serum. A renal biopsy revealed a necrotizing extracapillary proliferative glomerulonephritis with crescents, demonstrating MPO-ANCA-GN. Whereas, immunofluorescent staining documented granular deposition of immumoglobulin (Ig) G and C3 along the capillary wall and electron microscopy showed subepithelial deposits in the glomerular basement membrane demonstrating MN. Immunofluorescent staining of IgG subclass showed positive IgG1, IgG2, negative IgG3 and weak positive IgG4 suggested the possibility of malignancy-associated MN.

**Conclusion:**

Combination of MPO-ANCA-GN and MN are rare. Although the causal relationship has been suggested in some cases, we should consider all the possibilities including idiopathic MN and secondary MN associated with malignancy, drug use or infection.

## Background

Anti-neutrophil cytoplasmic antibody-associated glomerulonephritis (ANCA-GN) is usually characterized by necrotizing and crescentic glomerulonephritis without the deposition of immunoglobulin and complement, therefore, it is called pauci immune type. Whereas, immunoglobulin depositions have been sometimes observed [1] and very rare cases of membranous nephropathy (MN) and the concurrent ANCA-GN and have been suggested [2-6]. The precise mechanism of the combination of these two etiologies is not clear due to the limited numbers of the patients. Here we describe a case of myeloperoxidase (MPO)-ANCA-GN complicated with MN developed in 73-years-old male.

## Case presentation

**Figure 1:**
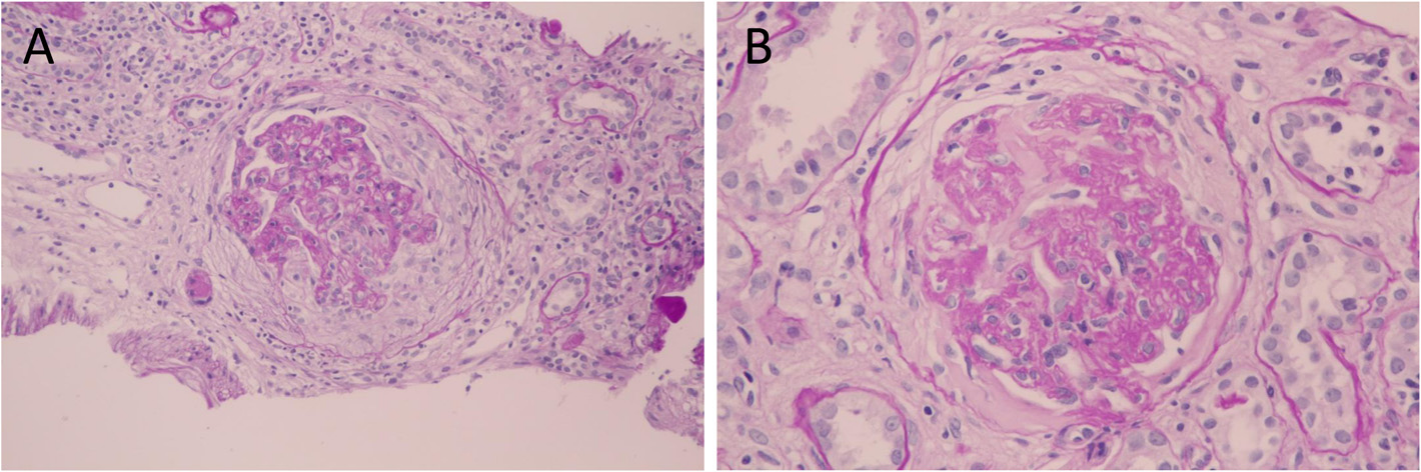
**Light microscopic findings.** Periodic acid-Schiff’s staining reveal a necrotizing extracapillary proliferative glomerulonephritis with crescent and many infiltrated mononuclear cells in the tubulointerstitium (**A**: original magnification × 200). A glomerulus with fibrocellular crescent and thickened glomerular basement membrane (GBM) (**B**: original magnification × 400). A glomerulus with fibrocellular crecent and thickened glomerular basement membrane (GBM) (Periodic acid-Schiff’s stain, original magnification × 400).

**Figure 2:**
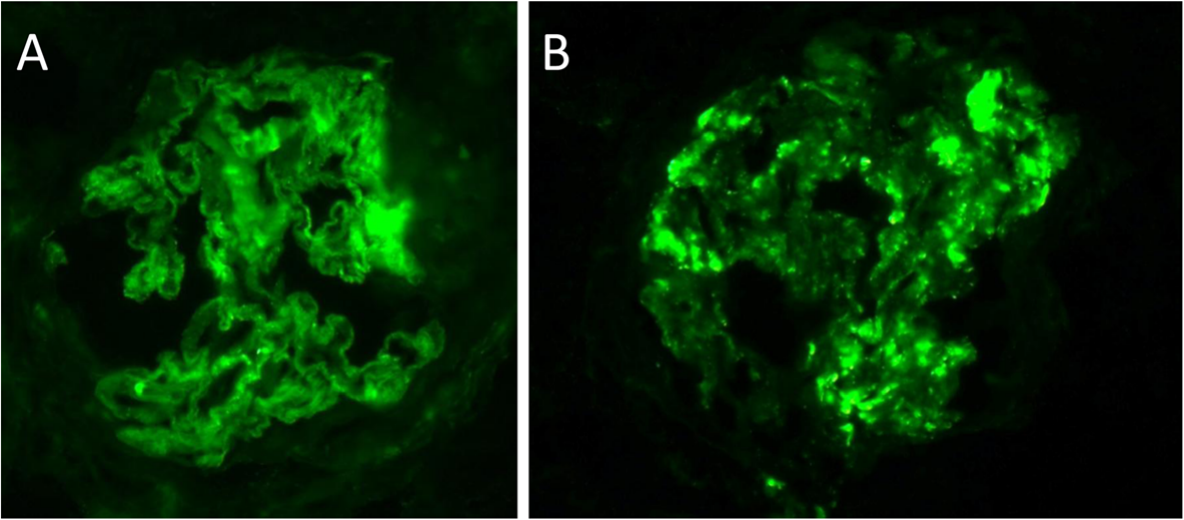
Immunofluorescent staining for IgG(A) and C3(B) reveals granular deposition in the glomerular capillary wall (original magnification × 400).

**Figure 3:**
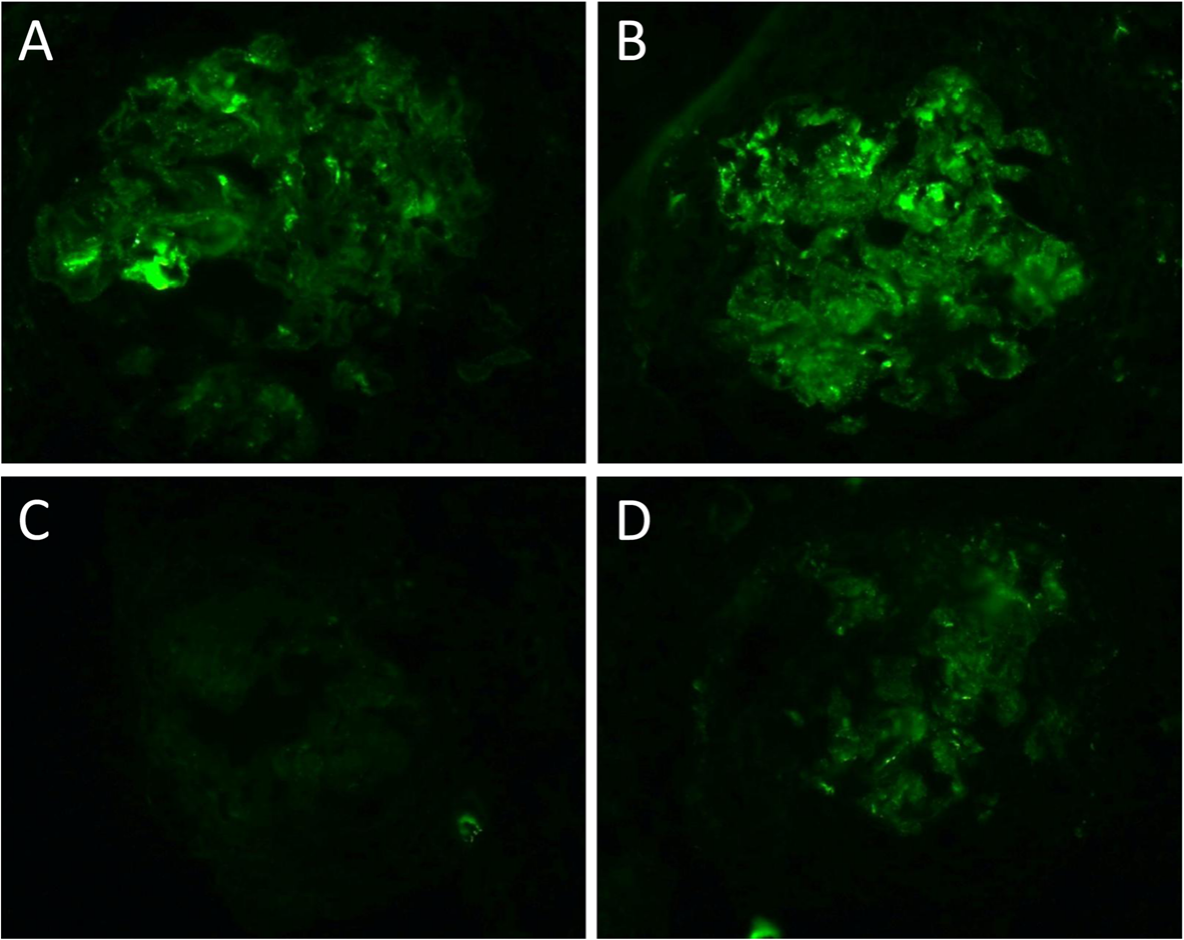
**Immunofluorescent staining for IgG subclass.** IgG1(**A**) and IgG2(**B**) reveals granular deposition in the glomerular capillary wall. IgG3(**C**) was negative and IgG4(**D**) was weak positive (original magnification × 400).

**Figure 4:**
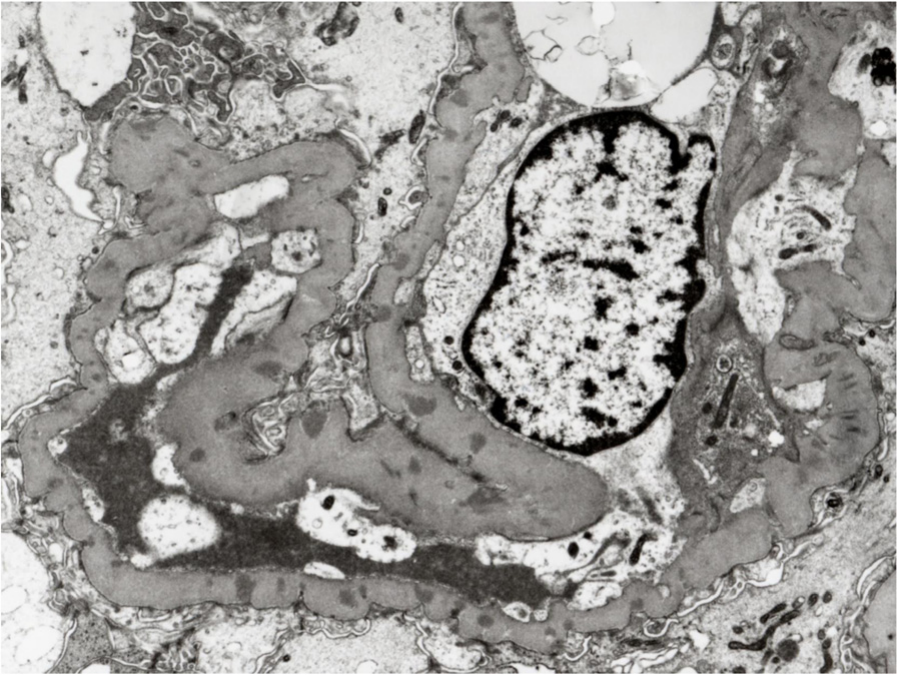
Electron microscopy shows electron dense deposits in subepithelial area of GBM (original magnification × 5000).

A 73-years-old male underwent a curative operation for a well-differentiated adenocarcinoma of the sigmoid colon. The tumor was (T2) with no lymphatic invasion (N0) and no distant metastasis (M0). Seven months later, he was referred to a nephrology division and admitted due to an exacerbating renal function and hypoalbuminemia without any clinical symptoms other than edema in the lower extremities. He had been diagnosed with hypertension, vasospastic angina and gastric ulcer for 11 years and treated with olmesartan medoxomil, nicorandil and roxatidine acetate. Proteinuria had been detected for 5 years (++ ~ +++). At the time of the pre-operative examinations, there was no specific symptoms. Serum creatinine was stable (1.2 mg/dl), C-reactive protein (CRP) was negative, proteinuria(++) and microhematuria was absent. Laboratory values at the time of referral are as follows: hemoglobin 10.1 g/dL, total protein 6.7 g/dL, albumin 3.1 g/dL, lactate dehydrogenase 208 U/L, blood urea nitrogen 59 mg/dL, creatinine 5.4 mg/dL, CRP 0.88 mg/dL, carcinoembryonic antigen (CEA) 4.1 ng/ml (negative), carbohydrate antigen(CA) 19–9 <1 U/ml (negative), MPO-ANCA >640 EU (positive), PR3-ANCA tested by ELISA <10 EU (negative), anit-glomerular besement membrane (GBM) antibody <10 EU (negative), anti nuclear antibody ×40 (normal limit < ×40), C3 97mg/dl, C4 59mg/dl. Urinalysis showed hematuria (+++): sediment red blood cells 40 /high power field and proteinuria 2.3 g/day. Chest CT scan revealed no specific findings. A renal biopsy was performed and documented a necrotizing extracapillary proliferative glomerulonephritis. There were 11 glomeruli, 5 with global sclerosis, 8 with mostly segmental crescents (5 fibro-cellular, 3 cellular) (Figure 1A, B). Routine immunofluorescent analysis revealed granular immumoglobulin G (IgG) and C3 deposition along the glomerular capillary wall (Figure 2A, B). IgA, IgM and fibrionogen were negative. Glomerular IgG subclass determined by immunofluorescent analysis showed positive IgG1 and IgG2, negative IgG3 and weak positive IgG4 deposition (Figure 3 A-D). Electron microscopy revealed electron-dense deposits in the subepithelial area of the GBM suggesting stage 3 MN (Figure 4).

3 week pulse therapy with methylprednisolone (500 mg for 3 days/week) followed by oral prednisolone (30 mg/day) decreased the levels of serum MPO-ANCA to normal range. Microhematuria was disappeared and CRP became negative, however, the levels of proteinuria and renal function did not improve. In a month, the antigenemia of the cytomegalovirus became positive without any clinical symptoms and was treated with valganciclovir. Therefore, prednisolone was tapered without adding immunosuppressants. Hemodialysis was initiated 6 months after the diagnosis of MPO-ANCA-GN. There was no recurrence of colon cancer and MPO-ANCA remained negative during the follow-up.

## Discussion

MN and concurrent MPO-ANCA-GN are very rare combination. It has been a question whether this is just a coincidence or there is a causal relationship. Recently, Hamamura et al. showed that MPO-ANCA-GN may cause MN-like lesions by demonstrating partial co-localization of MPO and IgG within the electron dense deposits [7]. There are also several characteristics observed in concurrent MN and ANCA-GN. Granular deposition of IgG and C3 along the GBM is similar, but IgG subclass deposition is different from predominant IgG4 pattern in idiopathic-MN and IgG1 and IgG4 depesition is reported in several cases [6,7]. This is compatible with the fact that the serum subclass of MPO-ANCA consisted mainly of IgG1 and IgG4 [8]. Besides, the electron microscopy findings show irregular dense deposits different from the global pattern of the idiopathic-MN. Due to the small numbers of the cases, further investigation is necessary for further understanding of the relationship between these two etiologies. In most cases, these two etiologies found concurrently, therefore, causal relationship is quite probable [2,5,7]. On the other hand, this may not explain all the cases, especially when MN and ANCA-GN diagnosed at different time course. In some cases, MPO-ANCA-GN was found during the remission of the MN [3,5]. These cases may be truly coincidental occurrence of the idiopathic-MN and MPO-ANCA-GN. Additionally, other causes such as malignancy-associated, infection-associated and drug-induced MN should be considered as well. In our case, MN and MPO-ANCA-GN were diagnosed simultaneously, but the long duration of the proteinuria and the colon cancer suggested the possibility of malignancy associated-MN which presumably preceded MPO-ANCA-GN at least to some extent. Electron microscopy findings also showed dense deposits of stage 3, relatively old lesion of MN. Additionally, predominant IgG1 and IgG2 deposition also suggested that MN in our case was secondary MN due to malignancy [9]. However, since we could not see the obvious improvement of proteinuria after the surgery of the colon cancer, we could not definitively diagnose that this case was malignancy associated-MN. Thus, concurrent MN and MPO-ANCA-GN possibly include different etiologies. Precise observation of these cases, especially the onset of the diseases, the analysis of IgG subclass in the glomerular deposition and the examination for the existence of the anti M-type phospholipase A2 receptor antibody which is demonstrated as the cause of idiopatic-MN also would help further understanding of these cases [10].

## Conclusions

Causal relationship has been suggested in the combination of MPO-ANCA-GN and MN in some cases, however, we should consider all the possibilities including malignancy, drug or infection associated MN as well as idiopathic MN. Besides, the analysis of the IgG subclass in the glomerular deposition may be of help.

## Consent

Written informed consent was obtained from the patient for publication of the study in the BMC Nephrology.

## Competing interests

The authors declare that they have no competing interests.

## Authors’ contributions

MS wrote the manuscript and was a treating physician for the patient. TF, NN, IN, YS, and RM were also treating physicians for the patient. YH, HO, KO performed the literature search and helped to write the manuscript. All authors read and approved the final manuscript.

## Pre-publication history

The pre-publication history for this paper can be accessed here:

http://www.biomedcentral.com/1471-2369/14/73/prepub
